# Supplementary material for: CAMP-negative Streptococcus agalactiae strains exhibited complete or partial chromosomal deletions of the CAMP-factor encoding gene cfb
Source: Microbiol Spectr. 2025 Apr 9;13(5):e03257-24. doi: 10.1128/spectrum.03257-24 (PMC12054153; doi:10.1128/spectrum.03257-24)
Supplement: Table S1 — The geographic distribution of current isolated ST862 GBS. [file spectrum.03257-24-s0001.docx]

Supplementary Table 1. The geographic distribution of current isolated ST862 GBS

| Source | Epidemiology | Country | Province | City | Isolation date | Capsular serotype | Reference |
| --- | --- | --- | --- | --- | --- | --- | --- |
| Pregnant women | Hospital | China | Guangdong | Guangzhou | 2017 | III | Cheng et al(1) |
| Pregnant women | Medical Center | South Korea |  | Beon-gil | 2016 | III-3 | Lee et al(2) |
| Pregnant women | Hospital | China | Guangdong | Shenzhen | 2020 -  2021 |  | Zhou et al(3) |
| Pregnant women | Hospital | China | Fujian | Xiamen | 2016 -  2018 | III | Yao et al(4) |
| Female patients | Hospital | China | Hainan | Haikou |  |  | Pu et al(5) |
| Pregnant women | Hospital | China | Fujian | Fuzhou | 2020 -  2021 | III (19 strains) and Ib (1 strains) | Liang et al(6) |
| Fish and Pork | Wet Markets | China |  | Hong Kong | 2016 -  2019 | III | Sapugahawatte et al(7) |
| Female patients | Community acquired | Singapore |  |  | 2015 | III | Data from [PubMLST](https://pubmlst.org/) |
| Female patients | Hospital | China | Hebei | Tangshan | 2022 |  |  |

**Reference**

1. Cheng Z, Qu P, Ke P, Yang X, Zhou Q, Lan K, He M, Cao N, Qin S, Huang X. 2020. Antibiotic Resistance and Molecular Epidemiological Characteristics of Streptococcus agalactiae Isolated from Pregnant Women in Guangzhou, South China. Can J Infect Dis Med Microbiol 2020:1368942.

2. Lee HT, Kim SY, Park PW, Ahn JY, Kim KH, Seo JY, Jeong JH, Kwoun WJ, Seo YH. 2019. Detection and genomic analysis of genital group B streptococcus in pregnant Korean women. J Obstet Gynaecol Res 45:69-77.

3. Zhou J, Zhang L, Zhang Y, Liu H, Xu K, Zhang B, Feng T, Yang S. 2023. Analysis of molecular characteristics of CAMP-negative Streptococcus agalactiae strains. Front Microbiol 14:1189093.

4. Yao Z, Jiayin W, Xinyi Z, Ling C, Mingyuan H, Simin M, Yayin L, Xinzhu L, Chao C. 2020. Identification of Group B Streptococcus Serotypes and Genotypes in Late Pregnant Women and Neonates That Are Associated With Neonatal Early-Onset Infection in a South China Population. Front Pediatr 8:265.

5. Pu W, Wang Y, Yang N, Guo G, Li H, Li Q, Ur Rehman N, Zheng L, Wang P, Han S, Zhou CC, Zheng J, Zeng J, Yuan J. 2020. Investigation of Streptococcus agalactiae using pcsB-based LAMP in milk, tilapia and vaginal swabs in Haikou, China. J Appl Microbiol 128:784-793.

6. Liang B, Chen H, Yu D, Zhao W, Cai X, Qiu H, Xu L. 2023. Molecular Epidemiology of Group B Streptococcus Isolates from Pregnant Women with Premature Rupture of Membranes in Fuzhou, China. Infect Drug Resist 16:269-278.

7. Sapugahawatte DN, Li C, Dharmaratne P, Zhu C, Yeoh YK, Yang J, Lo NWS, Wong KT, Ip M. 2022. Prevalence and Characteristics of Streptococcus agalactiae from Freshwater Fish and Pork in Hong Kong Wet Markets. Antibiotics (Basel) 11.
